# Development and consensus testing of quality indicators for geriatric pharmacotherapy in primary care using a modified Delphi study

**DOI:** 10.1007/s11096-022-01375-x

**Published:** 2022-04-05

**Authors:** Noriko Sato, Kenji Fujita, Kazuki Kushida, Timothy F. Chen

**Affiliations:** 1 School of Pharmacy, Faculty of Medicine and Health, The University of Sydney, Sydney, NSW Australia; 2 Departments of Clinical Pharmacology and Aged Care, Faculty of Medicine and Health, The University of Sydney, Kolling Institute, Sydney, NSW Australia; 3 Faculty of Pharmacy, Showa Pharmaceutical University, Tokyo, Japan

**Keywords:** Community pharmacy, Geriatric pharmacotherapy, Older people, Primary care, Quality indicators

## Abstract

*Background* Polypharmacy is associated with an increased risk of adverse drug events in older people. Although national guidance on geriatric pharmacotherapy exists in Japan, tools to routinely monitor the quality of care provided by community pharmacists are lacking. *Aim* To develop a set of quality indicators (QIs) to measure the quality of care provided by community pharmacists in improving geriatric pharmacotherapy in primary care in Japan, using a modified Delphi study. *Method* The development of QIs for the Japanese community pharmacy context followed a two–step process: national guidance review and consensus testing using a modified Delphi study. The latter involved two rounds of rating with a face–to–face meeting between the rounds. Ten experts in geriatric pharmacotherapy in primary care were recruited for the panel discussion. QIs were mapped to three key taxonomies and frameworks: the Anatomical Therapeutic Chemical (ATC) classification system, problems and causes of drug–related problems (DRPs) taxonomy and Donabedian’s framework. *Results* A total of 134 QIs for geriatric pharmacotherapy were developed. This QI set included 111 medicine specific indicators, covering medicines in 243 third–level ATC classifications. QIs were classified into the problem of treatment safety (80%) and causes of drug selection (38%) based on validated classification for DRPs. In Donabedian's framework, most QIs (82%) were process indicators. There were no structure indicators. *Conclusion* A set of 134 QIs for geriatric pharmacotherapy was rigorously developed. Measurement properties of these QIs will be evaluated for feasibility, applicability, room for improvement, sensitivity to change, predictive validity, acceptability and implementation issues in a subsequent study.

**Supplementary Information:**

The online version contains supplementary material available at 10.1007/s11096-022-01375-x.

## Impacts on practice

- A method for the development and evaluation of the face and content validity of quality indicators has been successfully used and may be applied in other settings and countries.
- The set of quality indicators may be used by pharmacists as a multidimensional assessment of geriatric pharmacotherapy in primary care.

## Introduction

The global population is aging and as a consequence, more people are living with multi–morbidity and the consequent polypharmacy [1–3]. Polypharmacy is associated with an increased risk of adverse drug reactions (ADRs) [4–6]. Thus, it makes sense to monitor older people taking multiple medicines in order to minimise the potential for medication–related harm.

Community pharmacists are in an ideal position to monitor the use of medicines for the people they serve as they are generally the last health care professionals individuals see before they initiate or continue to take their medicines. This monitoring role by community pharmacists in primary care directly aligns with a broader international trend towards the provision of professional pharmacy services, which came to prominence in the 1990s with the advent of “pharmaceutical care” [7]. Pharmaceutical care has been defined as ‘the pharmacist’s contribution to the care of individuals in order to optimise medicines use and improve health outcomes’ [8], highlighting the need for routine monitoring of the use of medicines within healthcare systems [9].

In line with this international trend, the Japanese Geriatrics Society published their first guideline for geriatric pharmacotherapy in 2005 [10], similar to the American Geriatrics Society which has published Beers Criteria in the US since 1991 [11]. The Japanese guidelines were updated in 2015 [12, 13] as were the Beers Criteria [14] and the Screening Tool of Older Person’s Potentially Inappropriate Prescriptions [15, 16]. These guidelines balance the potential benefits of using medicines with their associated risks. In 2018 the Ministry of Health, Labour and Welfare (MHLW) in Japan created guidance to reduce polypharmacy problems in collaboration with the Japanese Geriatrics Society [17]. The guidance is aimed at healthcare professionals including physicians, nurses and pharmacists, to ensure older people, particularly those aged 75 and above, use medicines in an optimal manner. Their scope was expanded in June 2019, specifically in relation to transitions of care between healthcare facilities and/or patients’ homes [18]. Consequently, the role of community pharmacists in the supply of medicines and medication management for older people at the risk of drug–related problems (DRPs) is increasing.

Although the guidance is designed to support healthcare professionals, specific instruction on how to utilise the content and monitor the quality use of medicines in community pharmacy is lacking. A well–recognised mechanism to measure care quality is via the use of quality indicators (QIs) across structure, process, or outcome domains [19]. QIs are usually defined with a denominator (the number of target population being measured) and a numerator (the number who have received the specific service), and measured as a percentage of correct actions (recommended care)[20]. Calculated QI scores indicate the quality of care and are monitored over time or for a specific period [21].

## Aim

The aim of this study was to develop QIs to measure the quality of care provided by community pharmacists in improving geriatric pharmacotherapy in primary care in Japan, using a modified Delphi study.

### Ethics approval

Approval was obtained from the social university general incorporated foundation ethical committee in Japan (SU1814, SU1912). Informed consent was obtained from all panellists.

## Method

**Fig. 1:**
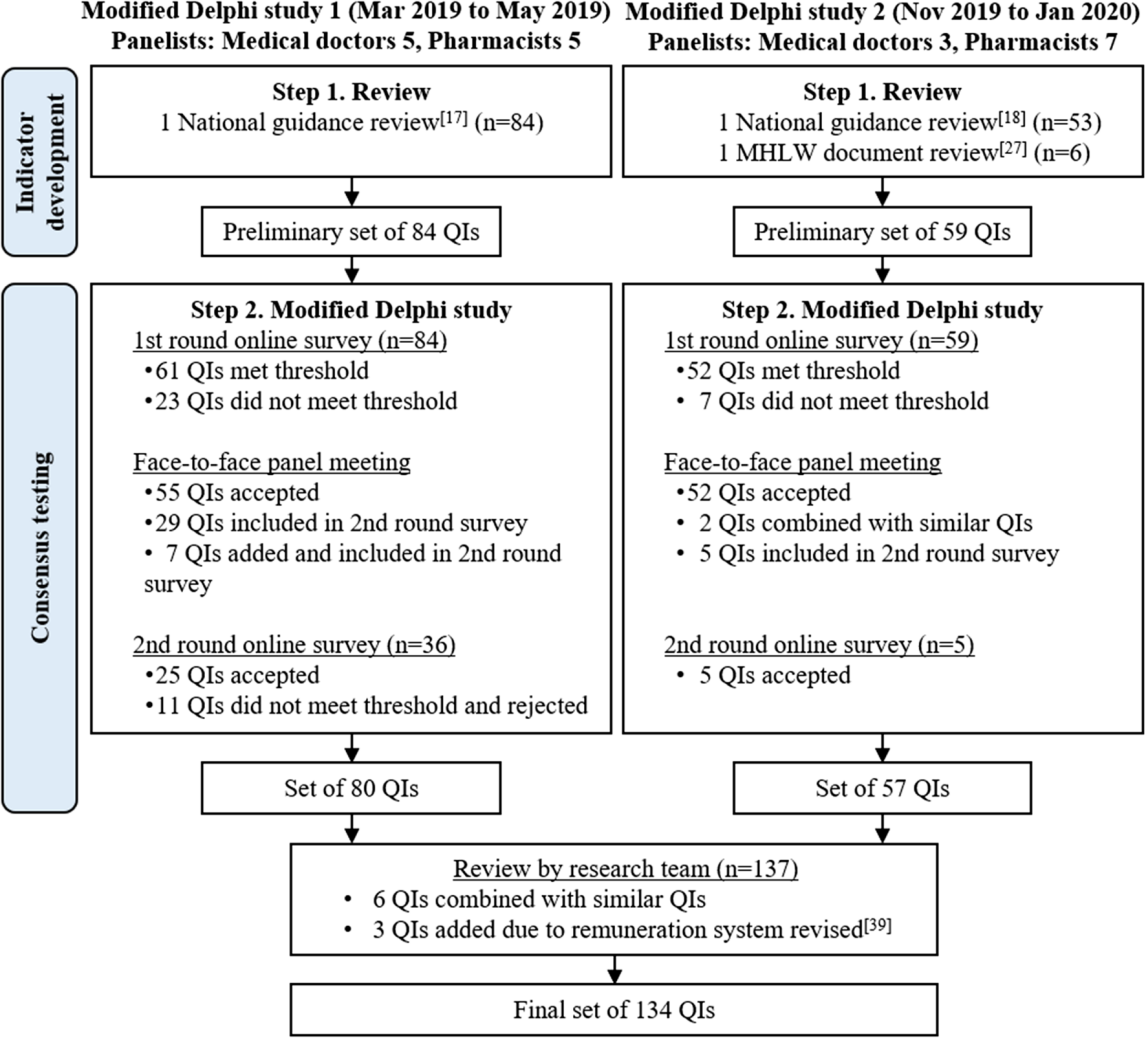
Study flow diagram

The development of QIs for the Japanese community pharmacy context followed a two–step process [22–24]. Two preliminary sets of QIs were developed following a comprehensive review. This was followed by a modified Delphi study for each set of QIs to achieve consensus [25]. The policy guidance documents were: ‘Guidance on Appropriate Medication for Elderly Patients (general) in 2018’ [17] and ‘Guidance on Appropriate Medication for Elderly Patients, particularly for the recuperation environment in 2019’ [18] (Fig. 1). Two separate sets of QIs were developed because the 2019 document was published whilst we were conducting a modified Delphi study of QIs based on the 2018 document. Hence two separate ethics approvals were obtained. The consensus data obtained from both documents were aggregated so that a comprehensive set of QIs for all therapeutic categories could be obtained. This study was reported in accordance with the consolidated criteria for reporting a Delphi study (CREDES) [26].

### Preparation of a preliminary set of QIs

A preliminary set of QIs was developed from each of the two evidence–based documents for geriatric pharmacotherapy [17, 18]. Additionally, a government document for remuneration of community pharmacy services was used to develop outcome indicators [27]. Principal researcher NS extracted recommendations from the aforementioned documents pertaining to quality use of medicines for older people and used this data to develop the preliminary sets of QIs [28–30]. A second researcher, KF, verified this process.

To undertake a comprehensive evaluation of the QIs, they were mapped to the following three key taxonomies and frameworks: (1) the Anatomical Therapeutic Chemical (ATC) classification system [31], (2) problems and causes of drug–related problems taxonomy (p–DRPs, c–DRPs, respectively) [32] and (3) Donabedian’s framework [19]. First, QIs were categorized into medicine specific indicators or general indicators, depending on whether the definition of QIs mentioned specific medicines. For instance, a QI about ‘laboratory monitoring of antidiabetics’ was classified as medicine specific indicators whereas a QI about ‘the assessment of swallowing function’ was categorised as general indicators. After this step, medicine specific indicators were classified according to the ATC code [31]. If QIs were related to more than one ATC code, they were mapped accordingly (e.g. for nonsteroidal anti–inflammatory drugs, ibuprofen is ‘M02AA13: musculo–skeletal system’ whereas acetylsalicylic acid is ‘N02BA01: nervous system’). Secondly, QIs were mapped to the classification system for DRPs developed by the Pharmaceutical Care Network Europe (PCNE) to determine the types of interventions which community pharmacists may undertake to resolve the causes of DRPs (Table 1) [32]. For example, a QI ‘QI–42 percentage of older patients taking warfarin who received an international normalised ratio monitoring’ pertained to ‘adverse drug event (possibly) occurring in p–DRPs (P2.1)’ and ‘no or inappropriate outcome monitoring in c–DRPs (C9.1)’. If QIs were related to more than one c–DRP, they were mapped accordingly. Lastly, QIs were also categorised into Donabedian’s framework of structure, process or outcome to identify the care type [19]. All classification was undertaken independently by NS and then verified by KF. The final mapping was discussed with all members of the research team (NS, KF, KK and TC).

**Table 1:** PCNE Classification for drug related problems in QIs

	P1.1 No effect of drug treatment despite correct use	P1.2 Effect of drug treatment not optimal	P1.3 Untreated symptoms or indication	P2.1 Adverse drug event (possibly) occurring	P3.1 Unnecessary drug treatment	N/A^b^	Total
***C1 Drug selection (n = 52)***							
C1.1 Inappropriate drug according to guidelines/formulary				13	1		14
C1.3 Inappropriate combination of drugs, or drugs and herbal medications, or drugs and dietary supplements				30			30
C1.4 Inappropriate duplication of therapeutic group or active ingredient				2			2
C1.5 No or incomplete drug treatment in spite of existing indication		2	2	1			5
C1.6 Too many different drugs/active ingredients prescribed for indication				1			1
***C2 Drug form (n = 0)***							
***C3 Dose selection (n = 14)***							
C3.1 Drug dose too low	1						1
C3.2 Drug dose of a single active ingredient too high				7			7
C3.3 Dosage regimen not frequent enough				4			4
C3.4 Dosage regimen too frequent				1			1
C3.5 Dose timing instructions wrong, unclear or missing				1			1
***C4 Treatment duration (n = 3)***							
C4.2 Duration of treatment too long				3			3
***C5 Dispensing (n = 6)***							
C5.2 Necessary information not provided or incorrect advice provided		1		5			6
***C6 Drug use process (n = 0)***							
***C7 Patient related (n = 17)***							
C7.1 Patient intentionally uses/takes less drug than prescribed or does not take the drug at all for whatever reason		3^a^		2^a^			5
C7.5 Patient takes food that interacts		1					1
C7.6 Patient stores drug inappropriately		1			1		2
C7.8 Patient unintentionally administers/uses the drug in a wrong way		3^a^		3^a^			6
C7.9 Patient physically unable to use drug/form as directed				2			2
C7.10 Patient unable to understand instructions properly		1					1
***C8 Patient transfer related (n = 1)***							
C8.1 Medication reconciliation problem		1					1
***C9 Other (n = 34)***							
C9.1 No or inappropriate outcome monitoring (incl. TDM)		1		10			11
C9.2 Other cause; ADR monitoring				23			23
N/A^b^						12	12
Total	1	14	2	108	2	12	139

*PCNE* Pharmaceutical Care Network Europe, *P* problems, *C* causes, *TDM* therapeutic drug monitoring, *ADR* adverse drug reaction.

^a^5 QIs were allocated to more than one c–DRPs.

^b^Outcome indicators which pertained to financial related outcome indicators (QI–123 to 134) were considered as not applicable (N/A).

### Consensus testing

A modified Delphi study, specifically the RAND/UCLA appropriateness method, was applied to combine evidence–based QIs with expert opinion [25]. It involved two rounds of rating with a face–to–face meeting between the rounds. For each modified Delphi study, a purposive selection of ten panellists with expertise in geriatric pharmacotherapy in primary care was recruited by e–mail or telephone (Supplementary Table 1). The modified Delphi studies were conducted between March 2019 and May 2019, and November 2019 and January 2020 (Fig. 1). All data at each stage were reviewed by the research team for feedback and editing before dissemination.

#### First round online survey

Panellists judged the face and content validity of each QI, using a 9–point scale (ranging from 1 “definitely not appropriate” to 9 “definitely appropriate”) with an opportunity to provide suggestions or modifications via *SurveyMonkey™*. This study defined appropriateness as “whether care described in the QIs must be provided in principle” and “whether a high QI score would be interpreted as a high–quality care” [33]. A QI with a median score of 7–9, without disagreement (i.e. at least 3 panellists scored 1–3, and at least 3 panellists 7–9) was judged as “appropriate” (i.e. median score ≥ 7, agreement 80%) [25]. The result from the first round and any additional comments made by panellists were de–identified and sent to all panellists one week before the panel meeting.

#### Face–to–face panel meeting

The expert panel meeting was conducted after the first round survey. QIs which did not achieve consensus as “appropriate” were discussed. Panellists were also invited to comment on ways to improve the comprehensiveness and accuracy of QIs which had reached consensus and propose other QIs to cover any perceived gaps. QIs which were re–worded and new potential QIs were included in the second round surveys. Panellists who were not able to attend the meeting in person provided written comments which were discussed at the expert panel meeting. The discussion at the panel meetings was digitally audio recorded.

#### Second round online survey

After the meeting, all panellists completed the same 9–point scale for evaluating QIs, as was used in the first round. Agreement was assessed using the same criteria as in the first round. After the second round, data from the two studies were aggregated and reviewed by the research team to assess overall comprehensiveness. The final result was sent to panellists for confirmation.

## Results

A set total of 134 QIs for geriatric pharmacotherapy in primary care was developed to assess the quality of care in community pharmacies (Fig. 1). The detailed description of QIs and results of consensus testing are provided in Table 2 and 3.

**Table 2:** Description of 134 quality indicators

No	QIs by therapeutic area	Numerator	Denominator
	*Sedative hypnotics/anxiolytics*		
1	ADR monitoring: Benzodiazepines	Number of those that were evaluated for ADRs (oversedation, cognitive decline, loss of motor function, falls, fractures)	Number of older people taking benzodiazepines
2	Guidance: Benzodiazepines	Number of those who received information about a benzodiazepine withdrawal syndrome	Number of older people taking benzodiazepines
3	Drug–drug interactions: Sedative hypnotics, anxiolytics	Number of those that were evaluated for drug–drug interactions	Number of older people taking the following medications:
			– Ramelteon & inhibitors of CYP1A2
			–﻿ Triazolam, alprazolam, brotizolam or suvorexant & inhibitors of CYP3A4
	*Antidepressants*		
4	Drug–disease interactions: Antidepressants	Number of those that were evaluated for drug–disease interactions (exacerbation of comorbidities)	Number of older people with epilepsy, narrow–angle glaucoma, cardiovascular disease or benign prostatic hyperplasia, taking antidepressants
5	Drug–drug interactions: Antidepressants	Number of those that were evaluated for drug–drug interactions (hemorrhage)	Number of older people taking the following medications:
			– Antidepressants & NSAIDs
			– Antidepressants & antiplatelets
6	ADR monitoring: TCAs	Number of those that were evaluated for ADRs (anticholinergic symptom, drowsiness, dizziness)	Number of older people taking TCAs
7	Drug–disease contraindications: TCAs, maprotiline	Number of those whose medical history of angle–closure glaucoma or recent myocardial infarction was checked	Number of older people taking TCAs or maprotiline
8	Drug–disease contraindications: TCAs, escitalopram	Number of those whose medical history of long QT syndrome was checked	Number of older people taking TCAs or escitalopram
9	ADR monitoring: Sulpiride	Number of those that were evaluated for ADRs (extrapyramidal symptoms)	Number of older people taking sulpiride
10	Medication appropriateness review: Sulpiride	Number of those who received appropriate monitoring (a renal function) by pharmacists and whose medications (use sulpiride ≤ 50 mg/day) were evaluated	Number of older people taking sulpiride
11	ADR monitoring: SSRIs	Number of those that were evaluated for ADRs (falls, gastrointestinal hemorrhage)	Number of older people taking SSRIs
12	Guidance: SSRIs	Number of those who received information about a SSRI withdrawal syndrome	Number of older people taking SSRIs
	*Drugs for BPSD*		
13	ADR monitoring: Antipsychotics	Number of those that were evaluated for ADRs (cognitive decline, extrapyramidal symptoms, falls, swallowing function, oversedation)	Number of older people taking antipsychotics
14	ADR monitoring: Yokukansan (Japanese traditional medicine)	Number of those that were evaluated for ADRs (pseudohyperaldosteronism)	Number of older people taking yokukansan (Japanese traditional medicine)
15	Drug–disease contraindications: Butyrophenones	Number of those whose medical history of Parkinson's disease was checked	Number of older people taking butyrophenones
16	Drug–disease contraindications: Atypical antipsychotics	Number of those whose medical history of diabetes was checked	Number of older people taking quetiapine or olanzapine
	*Antihypertensives*		
17	Medication appropriateness review: α–blockers	Number of those whose medications (discontinue α–blockers) were evaluated	Number of older people taking α–blockers in hypertension
18	Drug–drug interactions: CCBs	Number of those that were evaluated for drug–drug interactions	Number of older people taking the following medications:
			– Nisoldipine, felodipine, azelnidipine or nifedipine & inhibitors of CYP3A
19	Medication adherence: ACE inhibitors, ARBs 1	Number of those whose factors affecting medication adherence were listed and who received medication management services	Number of older people with poor medication adherence taking ARBs or ACE inhibitors
20	Medication adherence: ACE inhibitors, ARBs 2	Number of those who met the proportion of days covered threshold of 80% or more during the past 6 months	Number of older people taking ARBs or ACE inhibitors
21	Medication appropriateness review: Antihypertensives	Number of those whose medications (use CCBs, ARBs, ACE inhibitors or thiazide diuretics) were evaluated	Number of older people with hypertension, without CCBs, ARBs, ACE inhibitors or thiazide diuretics
22	Medication appropriateness: Antihypertensives	Number of those taking CCBs, ARBs, ACE inhibitors or thiazide diuretics	Number of older people taking antihypertensives
	*Antidiabetics*		
23	Medication appropriateness review: Sulfonylureas	Number of those whose medications (use DPP–4 inhibitors as an alternative drug) were evaluated	Number of older people taking sulfonylureas
24	Medication appropriateness: Sulfonylureas	Number of those without sulfonylureas	Number of older people taking antidiabetics
25	ADR monitoring: Sulfonylureas, self–injecting insulin	Number of those that were evaluated for ADRs (hypoglycemia)	Number of older people taking sulfonylureas or self–injecting insulin
26	Drug–drug interactions: Sulfonylureas, glinides	Number of those that were evaluated for drug–drug interactions	Number of older people taking the following medications:
			– Glimepiride, glibenclamide or nateglinide & inhibitors of CYP2C9
27	ADR monitoring: Biguanides	Number of those that were evaluated for ADRs (hypoglycemia, lactic acidosis, diarrhea)	Number of older people taking metformin
28	Medication appropriateness review: Thiazolidinediones	Number of those whose medications (discontinue pioglitazone) were evaluated	Number of older people with heart failure, taking pioglitazone
29	ADR monitoring: α–glucosidase inhibitors	Number of those that were evaluated for ADRs (ileus)	Number of older people taking α–glucosidase inhibitors
30	ADR monitoring: SGLT2 inhibitors	Number of those that were evaluated for ADRs (dehydration, unexplained weight loss, diabetic ketoacidosis, urogenital infection)	Number of older people taking SGLT2 inhibitors
31	Guidance: SGLT2 inhibitors	Number of those who received information about sick day management plan	Number of older people taking SGLT2 inhibitors
32	Medication appropriateness review: SGLT2 inhibitors	Number of those whose medications (discontinue diuretics) were evaluated	Number of older people taking the following medications:
			– SGLT2 inhibitors & diuretics
33	Laboratory monitoring: Antidiabetics	Number of those who received appropriate monitoring (HbA1c, blood glucose level) in pharmacies	Number of older people taking antidiabetics
	*Antihyperlipidemics*		
34	ADR monitoring: Statins	Number of those that were evaluated for ADRs (myalgia, digestive symptoms, new–onset diabetes)	Number of older people taking statins
35	Drug–drug interactions: Statins	Number of those that were evaluated for drug–drug interactions	Number of older people taking the following medications:
			– Fluvastatin & inhibitors of CYP2C9
			– Simvastatin or atorvastatin & inhibitors of CYP3A
36	Drug–drug contraindications: Statins	Number of those whose cyclosporine use was checked	Number of older people taking rosuvastatin or pitavastatin
37	Drug–drug interactions: Statins, fibrates	Number of those that were evaluated for drug–drug interactions	Number of older people with renal dysfunction taking the following medications:
			– Statins & fibrates
38	Medication appropriateness: Antihyperlipidemics	Number of those taking statins	Number of older people taking antihyperlipidemics
	*Anticoagulants*		
39	Drug–disease contraindications: DOACs	Number of those whose renal function (creatinine clearance > 30 ml/min) was checked	Number of older people taking DOACs
40	Drug–drug interactions: DOACs	Number of those that were evaluated for drug–drug interactions (hemorrhage)	Number of older people taking the following medications:
			– DOACs & antiplatelets
41	Drug–drug contraindications: Dabigatran	Number of those whose itraconazole use was checked	Number of older people taking dabigatran
42	Laboratory monitoring: Warfarin	Number of those who received appropriate monitoring (INR) in pharmacies	Number of older people taking warfarin
43	Guidance: Warfarin	Number of those who received information about food interactions with warfarin (foods rich in vitamin K)	Number of older people taking warfarin
	*Antiulcers*		
44	ADR monitoring: H2 blockers	Number of those that were evaluated for ADRs (cognitive decline)	Number of older people taking H2 blockers
45	Drug–drug interactions: PPIs	Number of those that were evaluated for drug–drug interactions	Number of older people taking the following medications:
			– Omeprazole or lansoprazole & inhibitors of CYP2C19
46	Medication appropriateness: PPIs	Number of those taking PPIs	Number of older people taking antiulcers
	*Antiinflammatories*		
47	ADR monitoring: Acetaminophen	Number of those that were evaluated for ADRs (liver dysfunction)	Number of older people taking acetaminophen overdose
48	Drug–drug interactions: NSAIDs 1	Number of those that were evaluated for drug–drug interactions (NSAIDs–induced ulcers)	Number of older people taking the following medications:
			– NSAIDs & antiplatelets
			– NSAIDs & anticoagulants
			– NSAIDs & glucocorticoids
49	Drug–drug interactions: NSAIDs 2	Number of those that were evaluated for drug–drug interactions (renal dysfunction, hyponatremia)	Number of older people taking the following medications:
			– NSAIDs & ARBs
			– NSAIDs & ACE inhibitors
			– NSAIDs & diuretics
50	Medication appropriateness review: NSAIDs 1	Number of those whose medications (use selective COX–2 inhibitors as an alternative drug) were evaluated	Number of older people with a medical history of peptic ulcers, taking NSAIDs
51	Medication appropriateness review: NSAIDs 2	Number of those whose medications (use PPIs or misoprostol) were evaluated	Number of older people taking NSAIDs for ≥ 3 months, without gastroprotection
52	Medication appropriateness: NSAIDs	Number of those taking PPIs or misoprostol	Number of older people taking NSAIDs for ≥ 3 months
	*Antimycobacterials/antivirals*		
53	ADR monitoring: Antibiotics/antivirals excreted by the kidney	Number of those that were evaluated for ADRs	Number of older people with renal dysfunction taking vancomycin, aminoglycosides, fluoroquinolones or aciclovir
54	Drug–drug contraindications: Carbapenems	Number of those whose valproate use was checked	Number of older people taking carbapenems
55	Drug–drug interactions: Fluoroquinolones	Number of those that were evaluated for drug–drug interactions (convulsion)	Number of older people taking the following medications:
			– Fluoroquinolones & NSAIDs
56	Guidance: Tetracyclines, fluoroquinolones	Number of those who received information about that drugs containing Al/Mg/Fe should be separated by at least 2 h	Number of older people taking the following medications:
			– Tetracyclines & drugs containing Al, Mg or Fe
			– Fluoroquinolones & drugs containing Al, Mg or Fe
	*Laxatives*		
57	ADR monitoring: Magnesium oxide	Number of those that were evaluated for ADRs (nausea, vomiting, hypotensive, bradycardia, muscle weakness, drowsiness)	Number of older people taking magnesium oxide
	*Anticholinergics*		
58	ADR monitoring: Anticholinergics	Number of those that were evaluated for ADRs (dry mouth, constipation, cognitive decline)	Number of older people taking anticholinergics
	*Antidementia drugs*		
59	ADR monitoring: Memantine 1	Number of those that were evaluated for ADRs (dizziness, drowsiness)	Number of older people with renal dysfunction taking memantine
60	ADR monitoring: Memantine 2	Number of those that were evaluated for ADRs (drowsiness)	Number of older people taking memantine in the morning or noon
61	Medication appropriateness review: Memantine	Number of those whose medications (memantine ≤ 1 mg/day) were evaluated	Number of older people with renal dysfunction taking > 1 mg/day of memantine
62	Medication appropriateness: Memantine	Number of those taking ≤ 10 mg/day of memantine	Number of older people with renal dysfunction, taking memantine
63	Guidance: Rivastigmine	Number of those who received information about that new patch should be put in a different place on their skin	Number of older people taking rivastigmine (transdermal patch)
64	ADR monitoring: Rivastigmine	Number of those that were evaluated for ADRs (skin symptoms)	Number of older people taking rivastigmine (transdermal patch)
65	ADR monitoring: ChEIs	Number of those that were evaluated for ADRs (agitation, restlessness, irritability)	Number of older people taking ChEIs
66	Drug–drug interactions: ChEIs 1	Number of those that were evaluated for drug–drug interactions	Number of older people taking the following medications:
			– ChEIs & NSAIDs
			– ChEIs & a medical history of peptic ulcers
67	Drug–disease interactions: ChEIs	Number of those that were evaluated for drug–disease interactions (palpitation, arrhythmia)	Number of older people with cardiovascular disease, asthma, COPD or extrapyramidal symptoms, taking ChEIs
68	Drug–drug interactions: ChEIs 2	Number of those that were evaluated for drug–drug interactions	Number of older people taking the following medications:
			– Donepezil & inhibitors of CYP3A4
			– Galantamine & inhibitors of CYP2D6
69	Drug–drug interactions: ChEIs 3	Number of those that were evaluated for drug–drug interactions (nausea, vomiting, bradycardia)	Number of older people taking the following medications:
			– ChEIs & cholinergics
			– ChEIs & other ChEIs for myasthenia gravis or glaucoma
70	Medication appropriateness review: ChEIs	Number of those whose medications (discontinue antipsychotics, TCAs, histamine receptor antagonists, anticholinergics for Parkinson disease) were evaluated	Number of older people taking the following medications:
			–﻿ ChEIs & antipsychotics
			– ChEIs & TCAs
			– ChEIs & histamine receptor antagonists
			– ChEIs & anticholinergics for Parkinson disease
71	Medication appropriateness: ChEIs	Number of those without anticholinesterases (antipsychotics, TCAs, histamine receptor antagonists, anticholinergics for Parkinson disease)	Number of older people taking ChEIs
72	Medication administration for those with dementia 1	Number of those whose drug use process (patient, their family, carer) was checked	Number of older people taking ChEIs or memantine
73	Medication administration for those with dementia 2	Number of those who received proper support on management of their medicine (the use of pill calendars or pillbox)	Number of older people with dementia taking ChEIs or memantine, without any support from families or carers
	*Osteoporosis drugs*		
74	Drug–disease contraindications: Bisphosphonates	Number of those whose esophageal disorders and inability (stand or sit upright for at least 30 min postdose) were checked	Number of older people taking bisphosphonates
75	Duplications: Bisphosphonates	Number of those whose intravenous bisphosphonate use (zoledronate) was checked	Number of older people taking oral bisphosphonates
76	Guidance: Bisphosphonates, denosumab	Number of those who received information about the importance of regular dental check–ups	Number of older people taking bisphosphonates or denosumab (6 monthly injection)
77	Laboratory monitoring: Denosumab	Number of those who received appropriate monitoring (severe hypocalcemia, the blood calcium test) in clinics within 3 months	Number of older people receiving denosumab (6 monthly injection)
78	Medication appropriateness review: Raloxifene, bazedoxifene	Number of those whose ADL (a long period of inactivity, sitting, or bed rest) was evaluated	Number of older people taking raloxifene or bazedoxifene
79	Treatment duration: Teriparatide	Number of those whose treatment duration of teriparatide (initiation and completed date) was checked	Number of older people taking teriparatide
80	Medication appropriateness review: Teriparatide	Number of those whose medications (discontinue bisphosphonates/ calcium/ vitamin D) were evaluated	Number of older people taking the following medications:
			– Teriparatide & bisphosphonates
			– Teriparatide & calcium
			– Teriparatide & vitamin D
81	Medication appropriateness: Teriparatide	Number of those without taking bisphosphonates, calcium or vitamin D	Number of older people taking teriparatide (self–injection)
82	Drug–drug interactions: Vitamin D	Number of those that were evaluated for ADEs (cognitive decline)	Number of older people taking the following medications:
			– Vitamin D & calcium
83	Medication appropriateness review: Alfacalcidol	Number of those whose medications (use alfacalcidol < 1 μg/day) were evaluated	Number of older people taking ≥ 1 μg/day of alfacalcidol
84	Medication appropriateness: Alfacalcidol	Number of those taking < 1 μg/day of alfacalcidol	Number of older people taking alfacalcidol
	*COPD drugs*		
85	Medication appropriateness review: Oral corticosteroids	Number of those whose medications (discontinue oral steroids) were evaluated	Number of older people with chronic stable COPD taking oral steroids
86	Medication appropriateness review: ICS/LABA	Number of those whose medications (use ICS/LABA) were evaluated	Number of older people with severe COPD (frequent exacerbationschronic), without ICS/LABA
87	Drug–disease contraindications: LAMAs	Number of those whose medical history of angle–closure glaucoma was checked	Number of older people taking LAMAs
88	Drug–disease interactions: LAMAs	Number of those that were evaluated for drug–disease interactions (worsening of dysuria)	Number of older people with benign prostatic hyperplasia, taking LAMAs
89	ADR monitoring: LABAs	Number of those that were evaluated for ADRs (hypermagnesemia, tachycardia, trembling in the hands, hypokalemia, sleep disorder)	Number of older people taking LABAs
90	Drug–disease interactions: LABAs	Number of those that were evaluated for drug–disease interactions (exacerbation of comorbidities)	Number of older people with hypertension, angina, hyperthyroidism, or diabetes, taking LABAs
91	Drug–drug interactions: LABAs	Number of those that were evaluated for drug–drug interactions	Number of older people taking the following medications:
			– Steroid inhalers or indacaterol & inhibitors of CYP3A4
92	ADR monitoring: Theophylline	Number of those that were evaluated for ADRs (theophylline toxicity)	Number of older people taking theophylline
93	Laboratory monitoring: Theophylline	Number of those who received appropriate monitoring (the blood concentration levels) in clinics within 6 months	Number of older people taking theophylline
94	Drug–drug interactions: Theophylline	Number of those that were evaluated for drug–drug interactions	Number of older people taking the following medications:
			– Theophylline & inhibitors of CYP1A2
95	Guidance: Steroid inhalers	Number of those who received information about that they gargle and rinse their mouth with water after using an inhaler	Number of older people using steroid inhalers
96	Guidance: Inhalers	Number of those whose inhaler techniques were evaluated	Number of older people using inhalers
	*Analgesics for cancer pain*		
97	ADR monitoring: NSAIDs	Number of those that were evaluated for ADRs (gastrointestinal hemorrhage, renal dysfunction)	Number of older people in palliative care taking NSAIDs
98	ADR monitoring: Opioids	Number of those that were evaluated for ADRs (oversedation)	Number of older people in palliative care taking opioids
99	Laboratory monitoring: Opioids	Number of those who received appropriate monitoring (a renal function) in pharmacies	Number of older people in palliative care taking morphine or codeine
100	Drug–drug interactions: Opioids 1	Number of those that were evaluated for drug–drug interactions (drug–induced extrapyramidal symptoms)	Number of older people in palliative care taking the following medications:
			– Opioids & prochlorperazine
101	Drug–drug interactions: Opioids 2	Number of those that were evaluated for drug–drug interactions (respiratory depression, dizziness, hypotension, oversedation)	Number of older people in palliative care taking the following medications:
			– Opioids & phenothiazines, barbiturates or benzodiazepines
			– Opioids & TCAs
			– Opioids & first–generation H1 antihistamines
102	Drug–drug interactions: Opioids 3	Number of those that were evaluated for drug–drug interactions	Number of older people in palliative care taking the following medications:
			– Oxycodone or fentanyl & inhibitors of CYP3A4
103	ADR monitoring: Antipsychotics	Number of those that were evaluated for ADRs (akathisia)	Number of older people in palliative care taking antipsychotics
104	ADR monitoring: Pregabalin	Number of those that were evaluated for ADRs (dizziness, drowsiness)	Number of older people with renal dysfunction in palliative care taking pregabalin
105	Pain management	Number of those whose pain intensity was checked	Number of older people in palliative care taking non–opioid analgesics or opioids
	*Other drugs*		
106	ADR monitoring: Digitalis	Number of those that were evaluated for ADRs (digitalis toxicity)	Number of older people taking > 0.125 mg/day of digoxin
107	Laboratory monitoring: Digitalis	Number of those who received appropriate monitoring (the blood concentration levels, electrocardiography) in clinics within 3 months	Number of older people taking > 0.125 mg/day of digoxin
108	Medication appropriateness: Digitalis	Number of those taking ≤ 0.125 mg/day of digoxin	Number of older people taking digoxin
109	Laboratory monitoring: Antiepileptics	Number of those who received appropriate monitoring (the blood concentration levels) in clinics within 3 months	Number of older people taking phenytoin or phenobarbital
110	Duplications: Drugs for topical use	Number of those whose overstock of the medicines at home were evaluated	Number of older people using topical drugs for pain or dry skin
111	Duplications: Drugs from the same medication class	Number of those whose therapeutic duplications were evaluated	Number of older people taking at least 2 medications from the same therapeutic group
	*General*		
112	Background information	Number of those whose background information (family, people living together, social services taken) was checked	Number of older people
113	Supplements or OTC medicines	Number of those whose current herbal/natural supplements or OTC medicines (consumptions, frequency) were checked	Number of older people
114	Swallowing function	Number of those whose swallowing function was evaluated	Number of older people
115	Laboratory monitoring: Renal function	Number of those whose renal function was evaluated	Number of older people
116	Vaccination: Influenza	Number of those with a record of the immunisation status for influenza	Number of older people
117	Vaccination: Pneumococcus	Number of those with a record of the immunisation status for pneumococcus	Number of older people
118	Medication administration	Number of those whose drug use process (patient, their family, carer) was checked	Number of older people
119	Transitional care	Number of those for which medication reconciliation was conducted	Number of older people who had a transitional care
120	Medication adherence: Unused medicines	Number of those whose unused medicines were arranged by pharmacists	Number of older people with poor medication adherence
121	Willingness to deprescribe	Number of those whose preferences towards deprescribing were evaluated	Number of older people
122	Medication administration: Medication frequency	Number of those taking medicines ≤ 3 times in a day	Number of older people
	*Remuneration for pharmacy services*		
123	Follow–up services for those with diabetes	Number of claims for community pharmacy services that pharmacists provided a followed–up service for people taking sulfonylureas or self–injecting insulin and reported it to a prescriber	N/A
124	Follow–up services for those using inhalers	Number of claims for community pharmacy services that pharmacists demonstrated correct inhaler technique and made a report to a prescriber	N/A
125	Medication management services	Number of claims for community pharmacy services that pharmacists provided medication management services for people with poor medication adherence	N/A
126	Provision of patients' information to other healthcare professionals	Number of claims for community pharmacy services that pharmacists shared patients' information to other healthcare professionals as required	N/A
127	Provision of appropriate drug information to patients/carers/prescribers	Number of claims for community pharmacy services that pharmacists shared patients' information to a prescriber if necessary, or drug information to patients/carers if new information becomes available	N/A
128	Use of unused medicines	Number of claims for community pharmacy services that pharmacists found unused medicines and adjusted the days of prescription	N/A
129	Change in medication regimen	Number of claims for community pharmacy services that pharmacists suggested a change in medication regimen and prescribers accepted the recommendations	N/A
130	Deprescribing medicines	Number of claims for community pharmacy services that pharmacists made a deprescribing recommendation to a prescriber and ≥ 2 medications were deprescribed for people taking ≥ 6 medications	N/A
131	Deprescribing recommendations	Number of claims for community pharmacy services that pharmacists made deprescribing recommendations for people taking ≥ 6 medications to a prescriber	N/A
132	Provision of health promotion activities	Pharmacy provided the community–level health promotion activities within a year	N/A
133	Review of patient satisfaction survey	Percentage of people who are satisfied with pharmacy services	N/A
134	Evaluation of contribution to the community they serve	The pharmacy received additional financial incentives for exceeding or meeting agreed quality metrics (e.g. provision of more than 60 home pharmaceutical services, more than 5 attendance in a local–level multidisciplinary meeting)	N/A

*ACE inhibitors* angiotensin converting enzyme inhibitors, *ADE* adverse drug event, *ADL* activities of daily living, *ADR* adverse drug reaction, *Al* aluminium, *ARBs* angiotensin II receptor blockers, *BPSD* behavioural and psychological symptoms of dementia, *CCBs* calcium channel blockers, *ChEIs* cholinesterase inhibitors, *COPD* chronic obstructive pulmonary disease, *CYP1A2* cytochrome P450 family 1 subfamily A member 2, *CYP2C19* cytochrome P450 family 2 subfamily C member 19, *CYP2C9* cytochrome P450 family 2 subfamily C member 9, *CYP2D6* cytochrome P450 family 2 subfamily D member 6, *CYP3A4* cytochrome P450 family 3 subfamily A, *DOACs* direct oral anticoagulants, *DPP–4 inhibitors* dipeptidyl peptidase 4 inhibitors, *ICS/LABA* a combination of inhaled corticosteroid and long–acting beta2 agonist, *Fe* iron, *INR* international normalised ratio, *LABAs* long–acting beta2–agonists, *LAMAs* long–acting muscarinic antagonists, *Mg* magnesium, *NSAIDs* nonsteroidal anti–inflammatory drugs, *OTC medicines* over–the–counter medicines, *PPIs* proton pump inhibitors, *SGLT2 inhibitors* sodium–glucose cotransporter–2 inhibitors, *SSRIs* selective serotonin reuptake inhibitors, *TCAs* tricyclic antidepressants.

**Table 3:** Classification of quality indicators and result of Modified Delphi studies

No	Third level of ATC code	p–DRPs	c–DRPs	Type	Unit	Modified	Round 1		Round 2	
						Delphi No	Median score	Agreement (%)	Median score	Agreement (%)
	*Sedative hypnotics/anxiolytics*									
1	N05B, N05C	P2.1	C9.2	P	%	D1	8.5	80	–	–
2	N05B, N05C	P2.1	C5.2	P	%	D1	8	90	–	–
3	N05B, N05C	P2.1	C1.3	P	%	D1	8.5	70	8	90
	*Antidepressants*									
4	N05A, N06A	P2.1	C9.2	P	%	D1	7.5	80	–	–
5	N05A, N06A	P2.1	C1.3	P	%	D1	9	80	–	–
6	N06A	P2.1	C9.2	P	%	D1	8	90	–	–
7	N06A	P2.1	C1.1	P	%	D1	8.5	90	–	–
8	N06A	P2.1	C1.1	P	%	D1	8.5	70	8	100
9	N05A	P2.1	C9.2	P	%	D1	8	70	8	90
10	N05A	P2.1	C3.2	P	%	D1	7.5	90	8.5	100
11	N06A	P2.1	C9.2	P	%	D1	8	70	8	90
12	N06A	P2.1	C5.2	P	%	D1	9	90	–	–
	*Drugs for behavioural and psychological symptoms of dementia*									
13	N05A	P2.1	C9.2	P	%	D1	8.5	80	–	–
14	Not available	P2.1	C9.1	P	%	D1	7	60	9	90
15	N05A	P2.1	C1.1	P	%	D1	9	90	–	–
16	N05A	P2.1	C1.1	P	%	D1	9	90	–	–
	*Antihypertensives*									
17	C02C	P2.1	C1.1	P	%	D1	8	90	–	–
18	C08C	P2.1	C1.3	P	%	D1	8	90	–	–
19	C09A, C09C, C09D	P1.2	C7.1/C7.8	P	%	D1	9	100	–	–
20	C09A, C09C, C09D	P1.2	C7.1/C7.8	O	%	D1	9	80	–	–
21	C02A, C02C, C02D, C02L, C03B, C03C, C03D, C07A, C09X	P1.2	C1.5	P	%	D1^c^	7, 7	70, 70	8, 7	90, 80
22	C02A, C02C, C02D, C02L, C03A, C03B, C03C, C03D, C07A, C08C, C08D, C09A, C09C, C09D, C09X, C10B	P2.1	C1.5	O	%	D1	7.5	60	8	80
	*Antidiabetics*									
23	A10B	P2.1	C1.1	P	%	D1	7.5	80	8	80
24	A10B	P2.1	C1.1	O	%	D1	7	70	8	90
25	A10A, A10B	P2.1	C9.2	P	%	D1	9	100	–	–
26	A10B	P2.1	C1.3	P	%	D1	8	80	–	–
27	A10B	P2.1	C9.2	P	%	D1	8.5	80	–	–
28	A10B	P2.1	C1.1	P	%	D1	8	70	8	100
29	A10B	P2.1	C9.2	P	%	D1	7.5	80	–	–
30	A10B	P2.1	C9.2	P	%	D1	9	80	–	–
31	A10B	P2.1	C5.2	P	%	D1	9	80	–	–
32	A10B	P2.1	C1.3	P	%	D1	8	80	–	–
33	A10A, A10B	P1.2	C9.1	P	%	D1	8.5	90	–	–
	*Antihyperlipidemics*									
34	C10A, C10B	P2.1	C9.2	P	%	D1	9	90	–	–
35	C10A, C10B	P2.1	C1.3	P	%	D1	7.5	80	–	–
36	C10A, C10B	P2.1	C1.3	P	%	D1	9	90	–	–
37	C10A, C10B	P2.1	C1.3	P	%	D1	8.5	70	8.5	100
38	C10A, C10B	P2.1	C1.1	O	%	D1	7.5	80	–	–
	*Anticoagulants*									
39	B01A	P2.1	C9.1	P	%	D1^c^	9, 8.5	90, 700	–	–, 90
40	B01A	P2.1	C1.3	P	%	D1	9	90	–	–
41	B01A	P2.1	C1.3	P	%	D1	9	90	–	–
42	B01A	P2.1	C9.1	P	%	D1	9	100	–	–
43	B01A	P1.2	C7.5	P	%	D1	9	100	–	–
	*Antiulcers*									
44	A02B	P2.1	C9.2	P	%	D1	8	90	–	–
45	A02B	P2.1	C1.3	P	%	D1	8.5	90	–	–
46	A02A, A02B, A03A, A03B, A16A	P2.1	C1.1	O	%	D1	8	70	8	80
	*Antiinflammatories*									
47	N02A, N02B	P2.1	C3.2	P	%	D1	8.5	90	–	–
48	M01A, N02B	P2.1	C1.3	P	%	D1	8.5	80	–	–
49	M01A, N02B	P2.1	C1.3	P	%	D1	7.5	90	–	–
50	M01A, N02B	P2.1	C1.1	P	%	D1	7.5	70	7.5	90
51	M01A, N02B	P2.1	C4.2	P	%	D1	8	90	–	–
52	M01A, N02B	P2.1	C4.2	O	%	D1	8	90	–	–
	*Antimycobacterials/antivirals*									
53	J01G, J01M, J01X, J05A	P2.1	C3.2	P	%	D1	8.5	80	–	–
54	J01D	P2.1	C1.3	P	%	D1	9	80	–	–
55	J01M	P2.1	C1.3	P	%	D1	9	80	–	–
56	J01A, J01M	P1.2	C5.2	P	%	D1	8.5	90	–	–
	*Laxatives*									
57	A06A	P2.1	C9.2	P	%	D1	8	80	–	–
	*Anticholinergics*									
58	A02B, A03A, A03B, A03F, C01B, G04B, M03B, N04A, N05A, N05B, N06A, R06A	P2.1	C9.2	P	%	D1	9	100	–	–
	*Antidementia drugs*									
59	N06D	P2.1	C3.2	P	%	D2	8	80	–	–
60	N06D	P2.1	C3.5	P	%	D2	8.5	80	–	–
61	N06D	P2.1	C3.2	P	%	D2	8	90	–	–
62	N06D	P2.1	C3.2	O	%	D2	8.5	70	8	100
63	N06D	P2.1	C5.2	P	%	D2	9	90	–	–
64	N06D	P2.1	C9.2	P	%	D2	9	100	–	–
65	N06D	P2.1	C9.2	P	%	D2	8.5	90	–	–
66	N06D	P2.1	C1.3	P	%	D2	7.5	80	–	–
67	N06D	P2.1	C9.2	P	%	D2	7.5	90	–	–
68	N06D	P2.1	C1.3	P	%	D2	8	80	–	–
69	N06D	P2.1	C1.3	P	%	D2	8	100	–	–
70	N06D	P2.1	C1.3	P	%	D2	8	100	–	–
71	N06D	P2.1	C1.3	O	%	D2	7.5	90	–	–
72	N06D	P2.1	C7.1/C7.8	P	%	D2	8.5	90	–	–
73	N06D	P1.2	C7.1/C7.8	P	%	D2	8	80	–	–
	*Osteoporosis drugs*									
74	M05B	P2.1	C7.9	P	%	D2	8.5	100	–	–
75	M05B	P2.1	C1.4	P	%	D2	8	90	–	–
76	M05B	P2.1	C5.2	P	%	D2	8	80	–	–
77	Not available	P2.1	C9.1	P	%	D2	7.5	90	–	–
78	G03X	P2.1	C1.1	P	%	D2	8	90	–	–
79	H05A	P2.1	C4.2	P	%	D2	8.5	90	–	–
80	H05A	P2.1	C1.3	P	%	D2^c^	8, 8	90, 80	–	–
81	H05A	P2.1	C1.3	O	%	D2^c^	8, 7.5	90, 80	–	–
82	A11C	P2.1	C1.3	P	%	D1	8	90	–	–
83	A11C	P2.1	C3.2	P	%	D1	8.5	90	–	–
84	A11C	P2.1	C3.2	O	%	D2	7.5	70	8	100
	*Chronic obstructive pulmonary disease drugs*									
85	H02A	P3.1	C1.1	P	%	D1	8.5	90	–	–
86	R03A, R03B, R03D	P1.2	C1.5	P	%	D2^b^	6.5, 6	50, 40	8	100
87	R03A, R03B	P2.1	C1.1	P	%	D2	9	100	–	–
88	R03A, R03B	P2.1	C9.2	P	%	D2	9	100	–	–
89	R03A, R03B	P2.1	C9.2	P	%	D2	8	90	–	–
90	R03A, R03B	P2.1	C9.2	P	%	D2	8	80	–	–
91	R03A, R03B	P2.1	C1.3	P	%	D2	7.5	80	–	–
92	R03D	P2.1	C9.2	P	%	D2	8	90	–	–
93	R03D	P2.1	C9.1	P	%	D2^e^	6.5, 7.5	50, 70	7.5, 8	90, 90
94	R03D	P2.1	C1.3	P	%	D2	8	90	–	–
95	R03A, R03B	P2.1	C1.3	P	%	D2	9	100	–	–
96	R03A, R03B	P1.2	C7.10	P	%	D2	8.5	100	–	–
	*Analgesics for cancer pain*									
97	M01A, N02B	P2.1	C9.1	P	%	D2	8	90	–	–
98	N02A	P2.1	C9.2	P	%	D2	8.5	90	–	–
99	N02A	P2.1	C9.1	P	%	D2^b^	8, 5	70, 30	8	90
100	N02A	P2.1	C1.3	P	%	D2	8	90	–	–
101	N02A	P2.1	C1.3	P	%	D2	7.5	90	–	–
102	N02A	P2.1	C1.3	P	%	D2	8.5	90	–	–
103	N05A	P2.1	C9.2	P	%	D2	8	100	–	–
104	N03A	P2.1	C3.2	P	%	D2	8	100	–	–
105	M01A, N02A, N02B	P1.1	C3.1	P	%	D2	8	80	–	–
	*Other drugs*									
106	C01A	P2.1	C3.2	P	%	D1	9	90	–	–
107	C01A	P2.1	C9.1	P	%	D1	6	50	8	80
108	C01A	P2.1	C3.2	O	%	D1	8	80	–	–
109	N03A	P2.1	C9.1	P	%	D1^a^	–	–	8	100
110	M02A	P3.1	C7.6	P	%	D1^a,a,c^	–	–	9, 8	90, 90
111	ALL*	P2.1	C1.4	P	%	D1	9	80	9	90
	*General*									
112	N/A	P2.1	C7.8	P	%	D2	8.5	90	–	–
113	N/A	P2.1	C1.3	P	%	D1	9	100	–	–
114	N/A	P2.1	C7.9	P	%	D1^a^	–	–	8.5	90
115	N/A	P2.1	C9.1	P	%	D1^a^	–	–	8.5	90
116	N/A	P1.3	C1.5	P	%	D1^a^	–	–	8.5	100
117	N/A	P1.3	C1.5	P	%	D1^a^	–	–	8.5	100
118	N/A	P2.1	C7.1/C7.8	P	%	D2	9	100	–	–
119	N/A	P1.2	C8.1	P	%	D1	9	100	–	–
120	N/A	P1.2	C7.6	P	%	D1^c^	9, 9	100, 100	–	–
121	N/A	P2.1	C1.6	P	%	D2	8	90	–	–
122	N/A	P2.1	C3.4	O	%	D1	9	80	–	–
	*Remuneration for pharmacy services*									
123	N/A	N/A	N/A	O	Number	D2^d^	–	–	–	–
124	N/A	N/A	N/A	O	Number	D2^d^	–	–	–	–
125	N/A	N/A	N/A	O	Number	D2	7	90	–	–
126	N/A	N/A	N/A	O	Number	D2	8	100	–	–
127	N/A	N/A	N/A	O	Number	D2	8	80	–	–
128	N/A	N/A	N/A	O	Number	D2	8	90	–	–
129	N/A	N/A	N/A	O	Number	D2	8	90	–	–
130	N/A	N/A	N/A	O	Number	D2	7	80	–	–
131	N/A	N/A	N/A	O	Number	D2^d^	–	–	–	–
132	N/A	N/A	N/A	O	Yes/No	D2	8	90	–	–
133	N/A	N/A	N/A	O	%	D2	8	100	–	–
134	N/A	N/A	N/A	O	Yes/No	D1	8.5	80	–	–

*ATC* The Anatomical Therapeutic Chemical, *p–DRPs* problems of drug–related problems, *c–DRPs* causes of drug–related problems, *N/A* Not applicable, *P* process, *O* outcome, *D1* Modified Delphi study 1, *D2* Modified Delphi study 2.

*All drugs used QIs were included.

^a^Quality indicators (QIs) added by panellists in the meeting.

^b^QIs combined by panellists in the meeting.

^c^QIs combined by researchers after the 2nd round.

^d^QIs added with the agreement of all panellists via e-mail after the 2nd round.

^e^QI assessed whether the care should be evaluated within 3 months or 6 months.

### Preparation of a preliminary set of QIs

A preliminary set of 137 QIs was developed from the national geriatric pharmacotherapy guidance documents [17, 18]. A further six potential QIs were developed based on a government detailing the remuneration of pharmacy services [27]. Hence, a preliminary set of 143 QIs was prepared for the consensus testing.

### Consensus testing

#### First round online survey

All panellists completed the survey. Of 143 preliminary QIs, 113 QIs were assessed as ‘appropriate’ and 30 QIs did not meet the threshold. No new QIs were proposed.

#### Face–to–face panel meeting

Eight panellists participated in each expert panel meeting, with comments in writing from two panellists who were absent (Supplementary Table 1). With agreement of all panellists who attended the meeting, 107 QIs were accepted without change or with minor rephrasing in response to the first round comments, or were combined with similar QIs. The remaining 34 QIs were discussed and modified to improve accuracy, and were included in the second round.

Additionally, 7 QIs were newly proposed based on panellists’ perspectives. QIs regarding ‘assessment of influenza and pneumococcus vaccination status’ were made in relation to a national immunisation programme [34–36]. QIs regarding ‘topical drugs for pain and dry skin’ were proposed to avoid oversupply since older people tend to store excess topical drugs [37, 38]. The assessment of functional status such as ‘swallowing function’ and ‘renal function’ was added by panellists, saying that ‘functional decline among older people should be monitored by community pharmacists as standard care’. Lastly, a QI on ‘laboratory monitoring of antiepileptic medicines’ was added.

#### Second round online survey

All panellists responded to the survey. Of 41 QIs, 30 QIs were assessed as ‘appropriate’. Eleven QIs did not meet the threshold (e.g. ‘use of angiotensin–converting enzyme inhibitors for hypertensive patients with recurrent aspiration pneumonia’ and ‘long–term stimulant laxative use’).

After both modified Delphi studies, the research team reviewed all accepted QIs to improve comprehensiveness. Twelve QIs which were deemed to be similar were combined into six QIs. Furthermore, three QIs pertaining to financial related outcome indicators were added by the research team with the agreement of all panellists, via e–mail. This process was undertaken to ensure the currency of the QI set because the government remuneration system for pharmacy services (for the period 2020 to 2022) was revised during the course of the study (e.g. number of claims for community pharmacy services that pharmacists demonstrated correct inhaler technique and made a report to a prescriber) [39]. As a result, 134 QIs were developed. The final result was sent to all panellists and confirmed by them.

### Characteristics of QIs

**Fig. 2:**
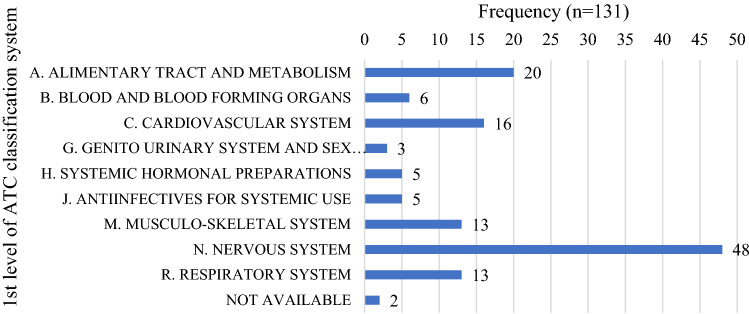
Number of QIs by the first level of ATC code

Key taxonomies and frameworks were used to understand the coverage of the QIs for medicines use in geriatric pharmacotherapy, acknowledging the multidimensional nature of responsible use of medicines in older persons. Of the 134 QIs developed in this study, the majority of QIs (n = 111, 83%) were medicine specific indicators. Some of them were allocated into more than one ATC code, resulting in 131 first–level ATC classifications (Fig. 2). The highest proportion of QIs pertained to nervous system (43%), followed by alimentary tract and metabolism (18%), cardiovascular system (14%) and respiratory system (12%). No QI pertained to dermatologicals, antineoplastic and immunomodulating agents, antiparasitic products, insecticides and repellents, sensory organs and various. Table 3 and Supplementary Table 2 present the third level ATC codes. Of 134, 122 QIs could be mapped to problems and causes associated with DRPs at the primary and sub–domain levels of the PCNE taxonomy. The remaining 12 QIs which pertained to financial related outcome indicators were not mapped (Table 1). The most common problems caused by DRPs at the sub–domain level were ‘adverse drug event (possibly) occurring (81%)’, followed by ‘effect of drug treatment not optimal (10%)’. For the causes of DRPs, QIs commonly mapped to ‘drug selection (39%)’, or ‘monitoring (25%)’. No QIs were found in the c–DRPs category of ‘drug form’ and ‘drug use process’. In total, the QIs were mapped to 139 c–DRP categories, as some QIs could be mapped to more than one c–DRP category. In terms of Donabedian’s framework, 110 QIs (82%) were process indicators and 24 QIs (18%) were outcome indicators (Table 3). No structure indicators were developed but it is noteworthy that the reporting structure indicators in Japan is mandatory (e.g. availability of pharmacy home visit services and pharmacy health promotion activities).

## Discussion

This seminal study described the development and consensus testing of a set of 134 QIs for geriatric pharmacotherapy, designed to evaluate the quality of care provided by community pharmacists in Japan in primary care. This QI set can be used for routine monitoring of the care provided by community pharmacists in optimising geriatric pharmacotherapy in primary care. Furthermore, the use of QIs could encourage community pharmacists to keep additional clinical patient records, ensuring that all important decisions are documented in line with the expansion of professional pharmacy services [40, 41].

Medicine specific indicators were widely distributed across 10 ATC categories at the first ATC level. The large number of ATC categories represented reflects the complexity of geriatric care, as this QI set targeted older people who have multiple conditions and medications. Most QIs could be mapped to medicines from the nervous system, alimentary tract and metabolism and the cardiovascular system, which aligns with QIs developed for other countries [42]. It follows that ATC categories with the greatest number of QIs would likely require significant opportunities for professional services from pharmacists. For example, when evaluating third level ATC codes, many QIs pertained to anti–dementia drugs (n = 15), blood glucose lowering drugs, excluding insulins (n = 11), antipsychotics (n = 10), other analgesics and antipyretics (n = 8) and, antidepressants (n = 8), all of which are known areas of importance for geriatric care in Japan [13] and other countries [42] (Supplementary Table 2). Likewise, there were no QIs for some ATC categories such as dermatologicals, indicating fewer opportunities for input from pharmacists.

Although most QIs were medicine specific indicators, there were 11 general indicators which were classified using the PCNE DRP taxonomy and Donabedian’s framework. Some of them appeared to be unique to the Japanese context. As an example, QIs for counselling about influenza and pneumococcus vaccination (QI–116,117) were unique in Japan where the administration of vaccines by pharmacists is not currently endorsed. In contrast, in Australia [43], the UK [44], the USA [45] and Canada [46], pharmacist vaccination programs exist yet no vaccine–related QIs pertaining to pharmacist vaccination exist. These QIs point to the importance of checking patients’ vaccine status and educating and/or reducing misconceptions about immunisation by community pharmacists. Furthermore, general indicators also included unique QIs such as ‘social services taken’ or ‘patients’ willingness to deprescribe’, which differed from existing general indicators that mainly focused on logistic issues such as medication reconciliation [47, 48]. That is, general indicators developed in this study might refer to the degree to which community pharmacists understand the patients’ background and can use this information to provide a more person–centred approach to medication management.

A large proportion of QIs was mapped to the c–DRP taxonomy including drug selection and monitoring related QIs. This may be explained by the fact that pharmacists play a critical role in resolving DRPs in these areas, considering the scope of pharmacy practice [41, 49, 50]. The majority of preventable DRPs are attributed to these categories [42]. On the other hand, no QI was included in the c–DRP category of ‘drug form’. Because a QI regarding ‘evaluation of swallowing function’ was developed, this QI could prompt pharmacists to find such a problem in patient counselling, if necessary, with pharmacists suggesting recommendations about drug form. Since the PCNE classification for DRPs is well–recognised and internationally used in medication management research, mapping QIs to this taxonomy provides an opportunity to compare QI scores between countries where the same taxonomy is used. Thus the quality of care can be assessed from multiple perspectives by stratifying QI results using this taxonomy.

Most QIs developed in this study were process indicators. This is not surprising and aligns with other data which shows that more than 90% of existing QIs for responsible use of medicines were process indicators [42, 51]. Indeed, since 2008 when the MHLW in Japan community pharmacy remuneration to generic–drug dispensing rates, performance–based payment models have expanded. Therefore, associations between process indicator scores and subsequent outcomes are expected to be evaluated.

Twelve of the 23 QIs which were mapped to outcomes pertained to financial outcomes (e.g. QI–123 follow–up services for those with diabetes), with the remainder aligning to medication appropriateness (e.g. QI–24 percentage of diabetic patients without sulfonylureas). It is noteworthy that whilst medication–related QIs which described adherence to the guideline may be considered as process indicators for physicians, they are considered outcome indicators for pharmacists. If pharmacists detect and reduce potentially inappropriate medications among older people by making a recommendation to a prescriber, adherence to the guideline could be improved. Indeed, a similar QI ‘percentage of cardiovascular patients with concomitant statin use’ has been used by community pharmacists in the Netherland as an outcome indicator [52].

We acknowledge that this study has some strengths and limitations. This study involved panellists with expertise in primary care and medication safety from different backgrounds including medical doctors and pharmacists. However, other healthcare professionals such as nurses were not included. Moreover, the QI characteristics were described using ATC classification system, but not all medicines are included in the ATC system (e.g. yokukansan: Japanese traditional medicine). In addition, the QI set was developed for the Japanese context as it was based on recommendations from national guidance similar to guideline recommendations. As geriatric pharmacotherapy guidelines can vary from country to country, these QIs may not be applicable to other countries. However, we believe that the concept and challenges for the appropriate use of medicines in older people are similar. Therefore, this QI set designed for Japanese pharmacies could also be of value to pharmacists in primary care in other countries.

## Conclusion

A set of 134 QIs for monitoring and evaluating geriatric pharmacotherapy by community pharmacists in primary care was rigorously developed. This QIs set could provide specific data to inform strategies to optimise geriatric patient care by community pharmacists in Japan. The measurement properties of QIs will be further evaluated for feasibility, applicability, room for improvement, sensitivity to change, predictive validity, acceptability and implementation issues.

## Supplementary Information

Below is the link to the electronic supplementary material.

Supplementary file1 (PDF 194 kb)
