# Supplementary material for: Development and consensus testing of quality indicators for geriatric pharmacotherapy in primary care using a modified Delphi study
Source: Int J Clin Pharm. 2022 Apr 5;44(2):517–38. doi: 10.1007/s11096-022-01375-x (PMC9007756; doi:10.1007/s11096-022-01375-x)
Supplement: Supplementary file 1 — Supplementary file1 (PDF 194 kb) [file 11096_2022_1375_MOESM1_ESM.pdf]

Supplementary materials

List of supplemental information:

- Supplementary Table 1. Characteristics of panellists
- Supplementary Table 2. Number of QIs by the third level of ATC code

Supplementary Table 1. Characteristics of panellists

No.	Panellists	Affiliation (Type of pharmacy)	Modified Delphi study 1 (Mar to May 2019)			Modified Delphi study 2 (Nov 2019 to Jan 2020)		
			1st round	Panel meeting	2nd round	1st round	Panel meeting	2nd round
1	Medical doctor	Associate professor of public university	〇	〇	〇	〇	〇	〇
2	Medical doctor	Private hospital	〇	×	〇	–	–	–
3	Medical doctor	Associate professor of private university	〇	〇	〇	–	–	–
4	Medical doctor	Representative of the Japan Geriatrics Society, auditor of Japan Association for Home Care Medicine, director of the Japanese academy of home care physicians, president of medical clinic	〇	〇	〇	〇	〇	〇
5	Medical doctor	Associate professor of public university, director of the Japanese academy of home care physicians, member of study group for appropriate medication for elderly patients	〇	〇	〇	〇	〇	〇
6	Pharmacist	Owner pharmacist (small chain)	〇	〇	〇	–	–	–
7	Pharmacist	Owner pharmacist (small chain)	〇	〇	〇	–	–	–
8	Pharmacist	Pharmacy manager (small chain)	〇	〇	〇	–	–	–
9	Pharmacist	Director, pharmacy department in private hospital	〇	〇	〇	〇	〇	〇
10	Pharmacist	Owner pharmacist (independent)<br>President, the local pharmaceutical association	〇	×	〇	〇	×	〇
11	Pharmacist	Pharmacy manager (independent)	–	–	–	〇	〇	〇
12	Pharmacist	Owner pharmacist (small chain)	–	–	–	〇	〇	〇
13	Pharmacist	General manager, community pharmacy company (big chain)	–	–	–	〇	〇	〇
14	Pharmacist	Manager, community pharmacy company (medium chain)	–	–	–	〇	×	〇
15	Pharmacist	Manager, community pharmacy company (big chain)	–	–	–	〇	〇	〇
		Total	10	8	10	10	8	10

Small-chain: 2-9 pharmacies, medium-chain: 10-99 pharmacies, big-chain: ≥ 100 pharmacies, 〇: attended, ×: absent, –: not applicable

Supplementary Table 2. Number of quality indicators by the third level of ATC code

The third level of the ATC classification system		Counts	(%)
A02A	ANTACIDS	2	(1.8)
A02B	DRUGS FOR PEPTIC ULCER AND GASTRO-OESOPHAGEAL REFLUX DISEASE (GORD)	5	(4.5)
A03A	DRUGS FOR FUNCTIONAL GASTROINTESTINAL DISORDERS	3	(2.7)
A03B	BELLADONNA AND DERIVATIVES, PLAIN	3	(2.7)
A03F	PROPULSIVES	2	(1.8)
A06A	DRUGS FOR CONSTIPATION	2	(1.8)
A10A	INSULINS AND ANALOGUES	3	(2.7)
A10B	BLOOD GLUCOSE LOWERING DRUGS, EXCL. INSULINS	12	(10.8)
A11C	VITAMIN A AND D, INCL. COMBINATIONS OF THE TWO	4	(3.6)
A16A	OTHER ALIMENTARY TRACT AND METABOLISM PRODUCTS	2	(1.8)
B01A	ANTITHROMBOTIC AGENTS	6	(5.4)
C01A	CARDIAC GLYCOSIDES	4	(3.6)
C01B	ANTIARRHYTHMICS, CLASS I AND III	2	(1.8)
C02A	ANTIADRENERGIC AGENTS, CENTRALLY ACTING	3	(2.7)
C02C	ANTIADRENERGIC AGENTS, PERIPHERALLY ACTING	4	(3.6)
C02D	ARTERIOLAR SMOOTH MUSCLE, AGENTS ACTING ON	3	(2.7)
C02L	ANTIHYPERTENSIVES AND DIURETICS IN COMBINATION	3	(2.7)
C03A	LOW-CEILING DIURETICS, THIAZIDES	2	(1.8)
C03B	LOW-CEILING DIURETICS, EXCL. THIAZIDES	3	(2.7)
C03C	HIGH-CEILING DIURETICS	3	(2.7)
C03D	POTASSIUM-SPARING AGENTS	3	(2.7)
C07A	BETA BLOCKING AGENTS	3	(2.7)
C08C	SELECTIVE CALCIUM CHANNEL BLOCKERS WITH MAINLY VASCULAR EFFECTS	3	(2.7)
C08D	SELECTIVE CALCIUM CHANNEL BLOCKERS WITH DIRECT CARDIAC EFFECTS	2	(1.8)
C09A	ACE INHIBITORS, PLAIN	4	(3.6)
C09C	ANGIOTENSIN II RECEPTOR BLOCKERS (ARBs), PLAIN	4	(3.6)
C09D	ANGIOTENSIN II RECEPTOR BLOCKERS (ARBs), COMBINATIONS	4	(3.6)
C09X	OTHER AGENTS ACTING ON THE RENIN-ANGIOTENSIN SYSTEM	3	(2.7)
C10A	LIPID MODIFYING AGENTS, PLAIN	6	(5.4)
C10B	LIPID MODIFYING AGENTS, COMBINATIONS	7	(6.3)
G03X	OTHER SEX HORMONES AND MODULATORS OF THE GENITAL SYSTEM	2	(1.8)
G04B	UROLOGICALS	2	(1.8)
H02A	CORTICOSTEROIDS FOR SYSTEMIC USE, PLAIN	2	(1.8)
H05A	PARATHYROID HORMONES AND ANALOGUES	4	(3.6)
J01A	TETRACYCLINES	2	(1.8)
J01D	OTHER BETA-LACTAM ANTIBACTERIALS	2	(1.8)
J01G	AMINOGLYCOSIDE ANTIBACTERIALS	2	(1.8)
J01M	QUINOLONE ANTIBACTERIALS	4	(3.6)
J01X	OTHER ANTIBACTERIALS	2	(1.8)
J05A	DIRECT ACTING ANTIVIRALS	2	(1.8)
M01A	ANTIINFLAMMATORY AND ANTIRHEUMATIC PRODUCTS, NON-STEROIDS	8	(7.2)
M02A	TOPICAL PRODUCTS FOR JOINT AND MUSCULAR PAIN	2	(1.8)
M03B	MUSCLE RELAXANTS, CENTRALLY ACTING AGENTS	2	(1.8)
M05B	DRUGS AFFECTING BONE STRUCTURE AND MINERALIZATION	4	(3.6)
N02A	OPIOIDS	8	(7.2)
N02B	OTHER ANALGESICS AND ANTIPYRETICS	9	(8.1)
N03A	ANTIEPILEPTICS	3	(2.7)
N04A	ANTICHOLINERGIC AGENTS	2	(1.8)
N05A	ANTIPSYCHOTICS	10	(9.0)
N05B	ANXIOLYTICS	5	(4.5)
N05C	HYPNOTICS AND SEDATIVES	4	(3.6)
N06A	ANTIDEPRESSANTS	9	(8.1)
N06D	ANTI-DEMENTIA DRUGS	16	(14.4)
R03A	ADRENERGICS, INHALANTS	9	(8.1)
R03B	OTHER DRUGS FOR OBSTRUCTIVE AIRWAY DISEASES, INHALANTS	9	(8.1)
R03D	OTHER SYSTEMIC DRUGS FOR OBSTRUCTIVE AIRWAY DISEASES	5	(4.5)
R06A	ANTIHISTAMINES FOR SYSTEMIC USE	2	(1.8)
	NOT AVAILABLE	2	(1.8)
Total		243*	

*QIs represented more than one code, resulting in 243 third levels of ATC classifications. ATC: The Anatomical Therapeutic Chemical
